# Dimensions of hospital resilience emphasized during the COVID‐19 pandemic response: A systematic review

**DOI:** 10.1002/hsr2.2300

**Published:** 2024-08-19

**Authors:** Golrokh Atighechian, Alireza Rahimi, Mohammad Sattari, Mahan Mohammadi

**Affiliations:** ^1^ Health Management and Economics Research Centre Isfahan University of Medical Sciences Isfahan Iran; ^2^ Health Information Technology Research Centre Isfahan University of Medical Sciences Isfahan Iran; ^3^ Centre for Environment and Population Health, School of Medicine and Dentistry Griffith University Brisbane Queensland Australia

**Keywords:** COVID‐19, disasters, hospital, pandemic, preparedness, resilience

## Abstract

**Objectives:**

Hospitals must maintain their effective operations during and after disasters. Due to the current increase in disasters, hospital resilience has drawn scholarly attention. This study aimed to review studies on the changes in the definition of hospital resilience after COVID‐19, build a conceptual framework for careful measurement, and identify the main dimensions of hospital resilience emphasized during the COVID‐19 pandemic.

**Design:**

The initial phase of this study was a systematic review of articles published before the COVID‐19 pandemic to extract the hospital resilience‐related dimensions for the second phase. The second phase involved text‐mining articles published both before and after the emergence of COVID‐19.

**Setting:**

In the systematic review phase, 12 databases were searched from 2006 to January 2020, including Scopus, Web of Science, MEDLINE through PubMed, Embase, ERIC, ProQuest, the Cochrane Library, Emerald, Springer, Science Direct/ELSEVIER, Google Scholar, and SID (for Persian language papers). Then, after COVID‐19, articles published in these databases between January 2020 and May 2022 were evaluated using text mining.

**Result:**

During the systematic phase, 17 out of 1530 papers published before COVID‐19 were synthesized to collect components of hospital disaster resilience. These identified components were the inputs for the text‐mining phase. The text mining on pre‐COVID papers resulted in six clusters, with the highest weight (0.65) belonging to general resilience and disaster preparedness, while in the post‐COVID text mining phase, including 70 papers, 8 clusters have been identified, with the highest weight cluster (0.78) focusing on the mental and psychological aspects of resilience among healthcare workers.

**Conclusion:**

Following the COVID pandemic, scholarly attention has shifted to the more personal dimensions of hospital resilience, including psychological resiliency. It seems necessary for policymakers to focus more on the individual and psychological resilience of hospital staff.

## 1 INTRODUCTION

Throughout human history, disasters have been unfortunate yet inevitable events that have significantly affected people's well‐being. These disasters have caused premature deaths, impaired quality of life and health status, and displaced many individuals.^1^ Natural and human‐induced disasters can impact affected communities and organizations, resulting in different levels of damage and associated costs.^2^, ^3^ Given the susceptibility of all organizations to disaster impacts, the concept of “organizational resilience” has been introduced to the glossary of disaster management as a new notion. Organizational resilience is an organization's ability to anticipate, be prepared for, respond to, and adapt to sudden disruptions to survive and prosper.^4^ One of the most active organizations in any disaster is the healthcare industry, and this definition has a vital application for this industry.

Hospitals are often front‐line healthcare service providers in disaster‐affected areas. However, during the initial surge of a disaster response, characterized by a sudden increase in demand for emergency medical services, hospitals can easily become overwhelmed with patients in acute conditions.^5^, ^6^, ^7^

Hospitals face multiple problems in terms of disaster preparedness and response. These challenges can affect infrastructure and technological capabilities, staff readiness, and coordination with other organizations, leading to resource limitations and ethical dilemmas. The resilience of hospital infrastructure and technological capabilities, including buildings, power supplies, and water systems, as well as the robustness of communication systems and information technology (IT) networks, are critical in coordinating response efforts. The challenges of resource limitations include shortages of medical supplies and equipment, insufficient physical capacity to handle the surge in patients, staff shortages and burnout, and financial constraints. Finally, hospitals may face ethical challenges in patient care, resource allocation, and staff safety.^8^, ^9^, ^10^, ^11^

The latest worldwide disaster, the COVID‐19 pandemic, has profoundly impacted hospital performance in different ways.^12^ This pandemic placed massive pressure on hospitals' scarce resources to address the high uncertainty related to this novel situation.^13^ As an example, during the first six months of the pandemic, nearly half of the hospital beds in the United States were occupied at 85% more than their capacity to meet the population's needs.^14^ Regardless of these big‐scale impacts of the COVID‐19 pandemic on hospitals' performance, less has been done to know about the dimensions of hospital resiliency that have been emphasized after this international disaster.

As a result, hospitals must have well‐researched plans that outline key elements and concepts. This is regarded as one of the highest priorities in emergency and disaster management.^15^, ^16^ Addressing these challenges in the form of resilience maintains operational capacity and ensures continuity in delivering essential health services during disasters.

The “hospital resilience” concept refers to a hospital's ability to withstand, absorb, and respond to disaster while maintaining critical functions, then recover to its original state or adapt to a new one.^16^, ^17^, ^18^, ^19^, ^20^ However, hospital post‐disaster recovery is a multifaceted and constantly evolving process requiring numerous requirements and resources.

Through a systematic approach, this study investigated the integration of hospital resiliency with disaster management. The research team noticed a dramatic increase in the number of papers on hospital resiliency, from an average of 40 papers each year before 2020 to an average of 300 papers since early 2020 (after the WHO announcement of the COVID‐19 outbreak), which indicates a significant increase in scholarly attention. This raises the question of whether there has been any modification to hospitals' definition of resilience.

Therefore, this paper aims to review studies on hospital resilience dimensions before and after the COVID‐19 pandemic, as an international disaster, to investigate how the most recent global disaster has affected these fundamental aspects of hospital resiliency. This will help build a conceptual framework for careful measurement and clarify the path for executive actions to guarantee optimal service provision.^21^

Research questions for this paper include the following:

- How has hospital resilience evolved following the COVID‐19 response?
- What components of hospital resilience have been emphasized following the COVID‐19 response?

## 2 MATERIALS AND METHODS

### 2.1 Study design

Authors conducted this study in two phases: the first phase involved a systematic review of articles published before COVID‐19, while the second phase involved text mining of articles published both before and after the pandemic. The first phase aimed to identify the concept and components of hospital resilience before COVID‐19. The second phase aimed to identify the differences in emphasis on hospital resilience components before and after COVID‐19, to examine the relationship between these components, and to determine the relative weight of each component.

1. Systematic Review Phase (Before COVID‐19)

**Figure 1:**
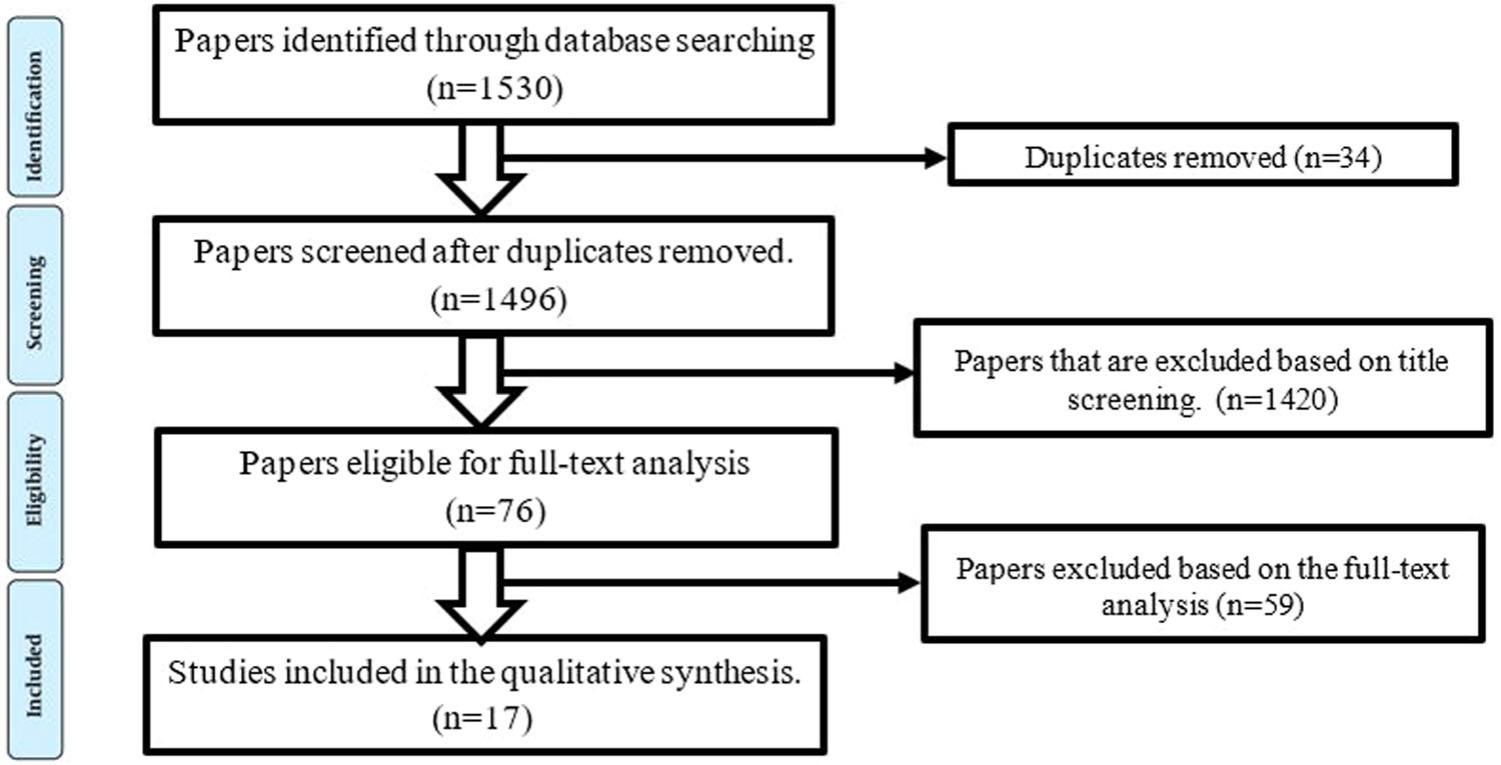
The screening process of articles obtained through searches before COVID‐19.

The Cochrane Handbook guided the systematic phase, and the findings were reported according to the PRISMA guidelines^22^, ^23^, ^24^ (Figure 1). The research team developed the protocol for systematic review, including the selection of databases, search terms, search limits, inclusion and exclusion criteria, creating data extraction forms, data analysis and synthesis froms, and reporting method. This phase aimed to identify the dimensions of hospital resilience in disasters before the COVID19.

**Figure 2:**
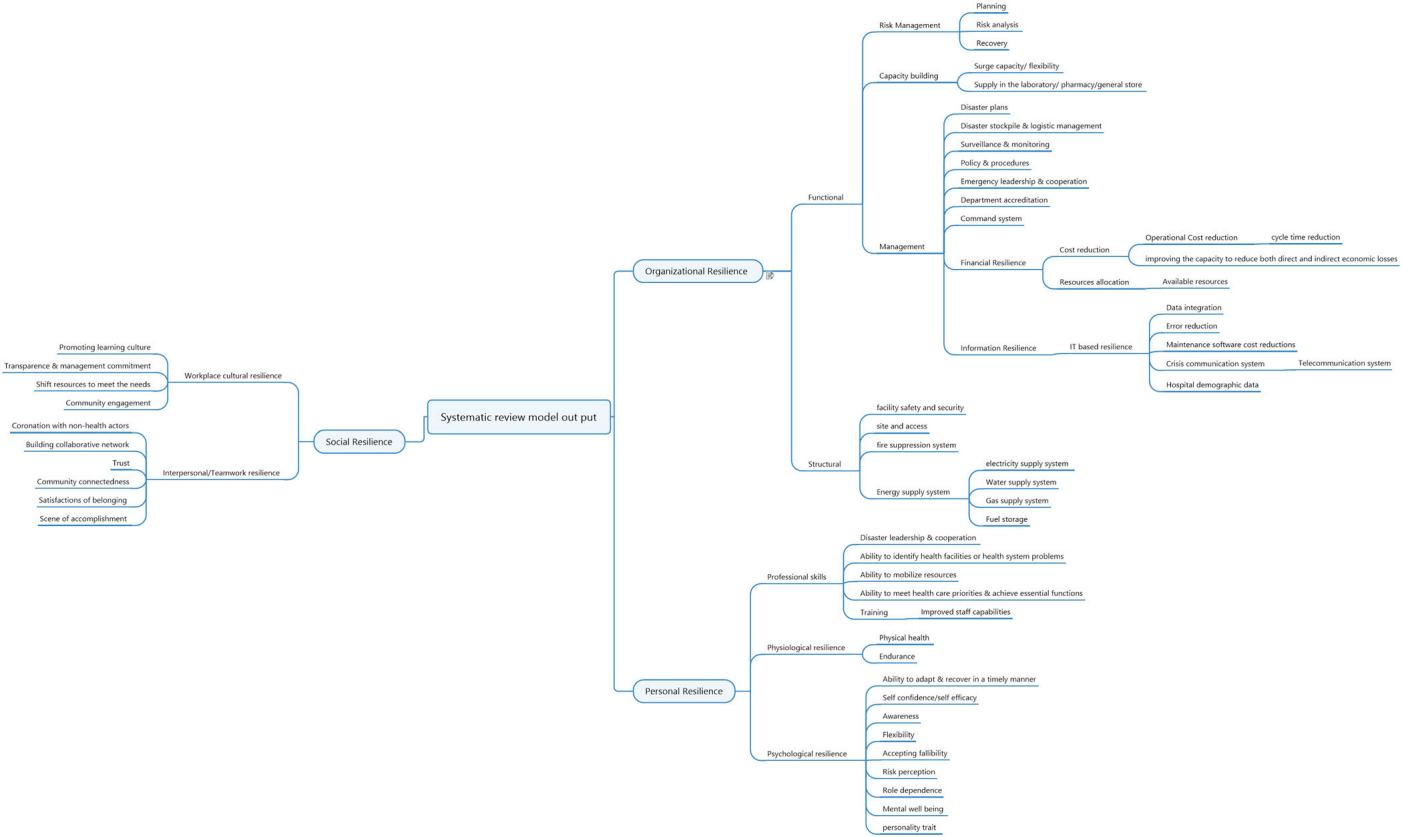
Dimensions/measures of hospital resilience before COVID‐19: Thematic results of systematic review.

**Table 1:** Search query.

Database	Search strategy	Result
**PubMed**	“resilienc*“[Title] AND (“hospital*“[Title] OR “health”[Title]) AND (“disaster*“[Title] OR “emergenc*“[Title] OR “crisis”[Title])	166
**Web of Science**	((TI = (Resilienc*)) AND TI = (Hospital* or health)) AND TI = (Disaster* or Emergenc* or crisis)	65
**ProQuest**	title(Resilienc*) AND title(Hospital* or health) AND title(Disaster* or Emergenc* or crisis)	140
**Embase**	(Resilienc*)/br AND ((Hospital* or health):ti) AND ((Disaster* or Emergenc* or crisis):ti)	57
**Scopus**	(TITLE (resilience) AND TITLE (hospital OR health) AND TITLE (disaster OR emergency OR crisis))	92
**ClinicalKey**	(Resilienc*)/br AND ((Hospital* or health):ti) AND ((Disaster* or Emergenc* or crisis):ti)	132
**Cochrane Library**	(Resilienc*):ti AND (Hospital* or health):ti AND (Disaster* or Emergenc* or crisis):ti	2
**ERIC**	Resilienc* and (Hospital* or health) and (Disaster* or Emergenc* or crisis) in TITLE	62
**Springer**	Resilienc* and (Hospital* or health) and (Disaster* or Emergenc* or crisis) in TITLE	225
**Science Direct/ELSEVIER**	Resilience and (Hospital or health) and (Disaster or Emergenc or crisis) in TITLE	395
**Google Scholar**	Resilience and (Hospital or health) and (Disaster or Emergenc or crisis)	189
**SID (for Persian language papers)**	Resilience and (Hospital or health) and (Disaster or Emergenc or crisis) in TITLE	5

**Table 2:** Critical appraisal template.

Country	Author(s)/year	Disaster type	Study objective	Study type/methods	Results/outputs of projects	Critical appraisal: 1. Quality of method and tools 2. Relevance to review questions

Concepts were disaster, emergency, crisis, resilience, resilience, resiliency, hospital, health, and hospital resilience.

- Inclusion Criteria

A) Formative: In 2006, on record, the number of papers using hospital resilinece related keywords more than doubled in the Scopus database, indicating a growing interest in this field. By studying these 22 articles, authors found that they were closely related to the topic of this paper. For this reason, authors chose a 13‐year timeframe for the study, from 2006 to January 2020 (pre‐COVID‐19 era). Additionally, authors included all Persian and English papers in the systematic phase of this study to explain the concept and identify the components of resilience in peer‐reviewed publications.
B) Conceptually: the study only included peer‐reviewed articles that directly addressed the research questions.

- Exclusion Criteria

A) Formative: In the first phase, authors excluded articles published before 2006 and those written in languages other than English and Persian. However, in the second phase (text mining phase), the Persian articles were excluded due to the incompatibility of text mining software (Python) in calculating the relationship between the extracted components of hospital resiliency in English and Persian languages.
B) Conceptual: the review excluded any study that did not discuss or indicate hospital resilience components. Therefore, studies that focused solely on organizational resiliency and were not in the health or hospital domain were excluded.

2. Sytematic Phase

- Selected databases
  The electronic databases searched included Scopus, Web of Science, MEDLINE through PubMed, Embase, ERIC, ProQuest, the Cochrane Library, Emerald, Springer, Science Direct/ELSEVIER, Google Scholar, and SID (for Persian language papers) (Table 1).
- Search Strategy
  This study utilized the following strategy in databases listed in Table 1, accessed through premium memberships provided by Isfahan University of Medical Sciences:
Resilienc*and(Hospital*orhealth)and(Disaster*orEmergenc*orcrisis)inTITLE
- Critical Appraisal
  GA and MM reviewed all the papers and used a data extraction template (Table 2). The papers were appraised based on the quality of their methods and tools, as well as the relevance of the study questions. From the 76 articles reviewed, the researchers selected 17 for the study.
  This study received the required ethics approval from the Isfahan University of Medical Sciences Research Ethics Committee, Iran, with ethics code no. IR. MUI. RESEARCH. REC.1397.067.
  Authors used the Cochrane Toolkit to assess the risk of bias in each study. In addition, the Hopkins‐WHO rigor scale was employed to evaluate bias and methodological quality in nonrandomised studies and to assess the quality of evidence and the strength of recommendations in healthcare. The Grading of Recommendations Assessment, Development, and Evaluation (GRADE) approach was used as a global standard.
  During the data synthesis, the collected evaluations were shared with the reviewers, and two workshops were organized to discuss the findings and reach a conclusive agreement. Continues feedback was sought for specific ambiguous domains before producing the final report, Figure 2 illustrate the extracted themes from included papers in systematic review phase.

3. Text Mining Phase (Before and After COVID‐19)
  The authors employed a two‐step text mining method for the periods before and after COVID‐19. First, the identified hospital resilience component from 17 papers from the pre‐COVID phase were analyzed. Then, for the post‐COVID‐19 period, articles published between January 2020 and May 2022 were evaluated using the text mining method. This stage examined the hospital resilience components, their relative weights, and their interconnections before and after COVID‐19. The method consist of six parts: pre‐processing, keyword extraction, text graphing, edge weight determination, clustering using the k‐means method, and creating graphs based on clusters. The databases and search strategy were similar to those used in the systematic review phase. The articles included in the systematic review phase were incorporated into the text mining phase (*N* = 17). Additionally, after comprehensive search and following the PRISM guidelines, 70‐screened articles published after COVID‐19 were included in the text mining phase.

4. Text Mining Steps

- Preprocessing
  Text involves cleaning the data and preparing it for input into the model. Noise in text data comes in many forms, including emotions, and punctuation. The first step is to identify sentences and extract less meaningful words from them. This includes removing stop words, such as symbols, numbers, general words, and words lacking any semantic value. Additionally, root words are identified and verbs (prefixes and suffixes) are eliminated. Python 3.11.4 was used for the pre‐processing procedure, utilizing various algorithms and libraries. Authors employed the most advanced library available, Spacy, for this task.
- Keyword extraction is the second step, performed
  Using the Term Frequency–Inverse Document Frequency (TF‐IDF) method. To obtain the TF‐IDF coefficient, each term should be calculated separately. The TF is the frequency of a word divided by the total number of words in the content, while the IDF is the logarithm of the total number of documents divided by the number of document containing desired word. The TF and IDF values are then multiplied to yield the keyword's weighted frequency, the formulas are as follows:
  TF = the division of the number of repetitions of the word by the total number of words in the content.
  IDF = the logarithm dividing the total number of contents by those containing the desired word.
$${\mathrm{Tf}-\mathrm{IDF}}_{\mathrm{xy}}={\mathrm{Tf}}_{\mathrm{xy}}*\log(\frac{N}{{\mathrm{df}}_{x}})$$
${\mathrm{Tf}}_{\mathrm{xy}}$ number of repetitions of word x in text y
${\mathrm{df}}_{x}$ is the number of documents that have the word x
*N* is the total number of documents
  In the Graph representation step, the text is represented as a graph G = (V, E), where V is the set of vertices and E is the edges.
  Each vertex corresponds to a token extracted in the previous step. Edges are created when tokens are used together in a sentence. If tokens i and j appear together in a sentence, they form E_ij._ For example, in this graph, Keywords such as "planning" and "risk analysis" serve as vertices. An edge between "planning" and "risk analysis" indicates that these are used together in a sentence, establishing a connection between them.
- Determining the weight of the edge
  The adjacency matrix for the text graph is created to assign weights to the edges. Edge weights are based on the number of times nodes co‐occur in the same sentence. This weight is calculated using the following formula:
$$W_{c}(\mathrm{ij})=\frac{\mathrm{freq}(\mathrm{ij})}{\mathrm{freq}\left(i\right)+\mathrm{freq}\left(j\right)-\mathrm{freq}(\mathrm{ij})}$$
  *freq(i)*: the number of repetitions of the word *i*
*freq(j)*: the number of repetitions of word *j*
*freq(i,j)*: the number of co‐occurrences of words *i* and *j* in a sentence
- Clustering based on the k‐means method
  One of the simplest and fastest clustering algorithms is K‐means. The parameter k in the algorithm specifies the number of clusters to obtain. Initially, K data points are selected as the cluster centers. This distances between the remaining data points is assigned to the cluste with the closest center. This process is repeated until the clusters remain unchanged. Here is a detailed explanation of the K‐means algorithm:
  input: k categories and n data characteristics.
  The result is k categories with data that are distinct from one another and closely related to one another in terms of similarity.
  Data k is selected to serve as the cluster's center.
  The third through fifth steps are repeated until the clusters remain the same.
  We use cluster centers as our reference to calculate the distances between the remaining data.
  Each cluster is filled with the data closest to its center.
  Each cluster's mean is now considered the cluster's new center.
  In approach case, k vertices are selected as the central nodes of the clusters. The similarity of the remaining vertices to these central nodes is measured based on the edge weight. Each node then assigned to the cluster with which it has the highest similarities.
- Creating a graph based on clusters

When creating a graph based on clusters, clusters are treated as nodes. The average weight between nodes in two clusters is considered the edge between those clusters.

## 3 RESULTS

### 3.1 Systematic review phase before COVID‐19

a) Characteristics of selected studies
  Appendix 1 outlines the characteristics of the selected papers. This table shows that most 17 studies focused on various types of disasters, including both natural and human‐induced disasters, whereas only three concentrated on one disastrous event, such as earthquakes. Among the selected papers, 10 followed qualitative approach, while seven were based on quantitative approaches. From the selected literature, practical research on hospital resilience has predominantly concentrated on recognizing the qualities that contribute to hospitals resilient.
b) Dimensions/measures of hospital resilience (results of systematic review)

The study identified three main themes contributing to hospital resilience following a systematic review. The first theme, organizational resilience, comprises both functional and structural resilience.^25^, ^26^, ^27^, ^28^, ^29^, ^30^, ^31^, ^32^, ^33^, ^34^, ^35^ The second theme, personal resilience, encompasses professional skills, psychological resilience, and physiological resilience.^36^, ^37^ The third theme, social resilience, includes workplace cultural and teamwork/interpersonal resilience (Figure 2).^38^, ^39^, ^40^

### 3.2 Text mining before COVID‐19

**Figure 3:**
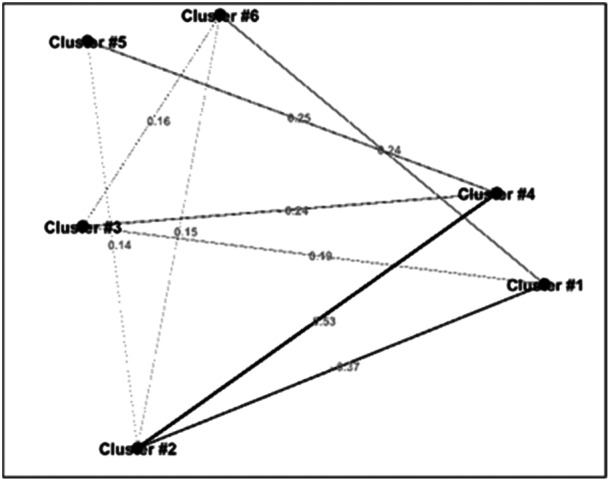
Relationship between clusters in pre‐COVID‐19 studies.

At this phase, authors classified the results into six clusters (Table 3). Cluster six, with the highest weight (weight = 0.65), focuses primerily on the general concepts of resilience, disaster preparedness, and disaster management. The results also revealed that, before COVID‐19, studies had given the least weight to individual resilience, including mental and psychological aspects, and functional resilience (cluster 1). The results also revealed a strong relationship between clusters 2 and 4 (w = 0.53; Figure 3).

**Table 3:** Clustering and weighting of findings from text mining before COVID‐19.

**Cluster 1, Weight: 0.42** − Mental health training − Mental health − Mental health unadjusted − Anxiety score − Psychological stress − Self confidence − Flexibility − Personality trait − Awareness	**Cluster 2, Weight: 0.53** − Health emergency preparedness − Emergency medical treatment − emergency training − Emergency services − Emergency leadership − Emergency preparedness − Emergency preparedness measures − Surveillance
**Cluster 3, Weight: 0.44** − Care provider organizations − Health leaders' efforts − Health care workers − Hospital disaster managers − Health manpower − Health manpower management − Health care provider − Improved staff capabilities − Healthcare priorities	**Cluster 4, Weight: 0.39** − Resilience performance − Error reduction − Average treatment time − Patient arrival rate − Efficient health responses − Health facilities − Hospital demographic data − Data integration
**Cluster 5, Weight: 0.65** − Disaster preparedness attributes − Preparedness attributes − Organizational resilience − Organizational resilience potential − Disaster resilience indicators − Disaster resilience frameworks − Resilience indicators − Disaster resilience assessment − Disaster management	**Cluster 6, Weight: 0.56** − Hospital functionality − Hospital disaster resources − Hospital functionality index − Hospital disaster plan − Hospital disaster resilience − Hospital disaster capacity − Hospital disaster preparedness

### 3.3 Text mininig after COVID‐19 began

**Figure 4:**
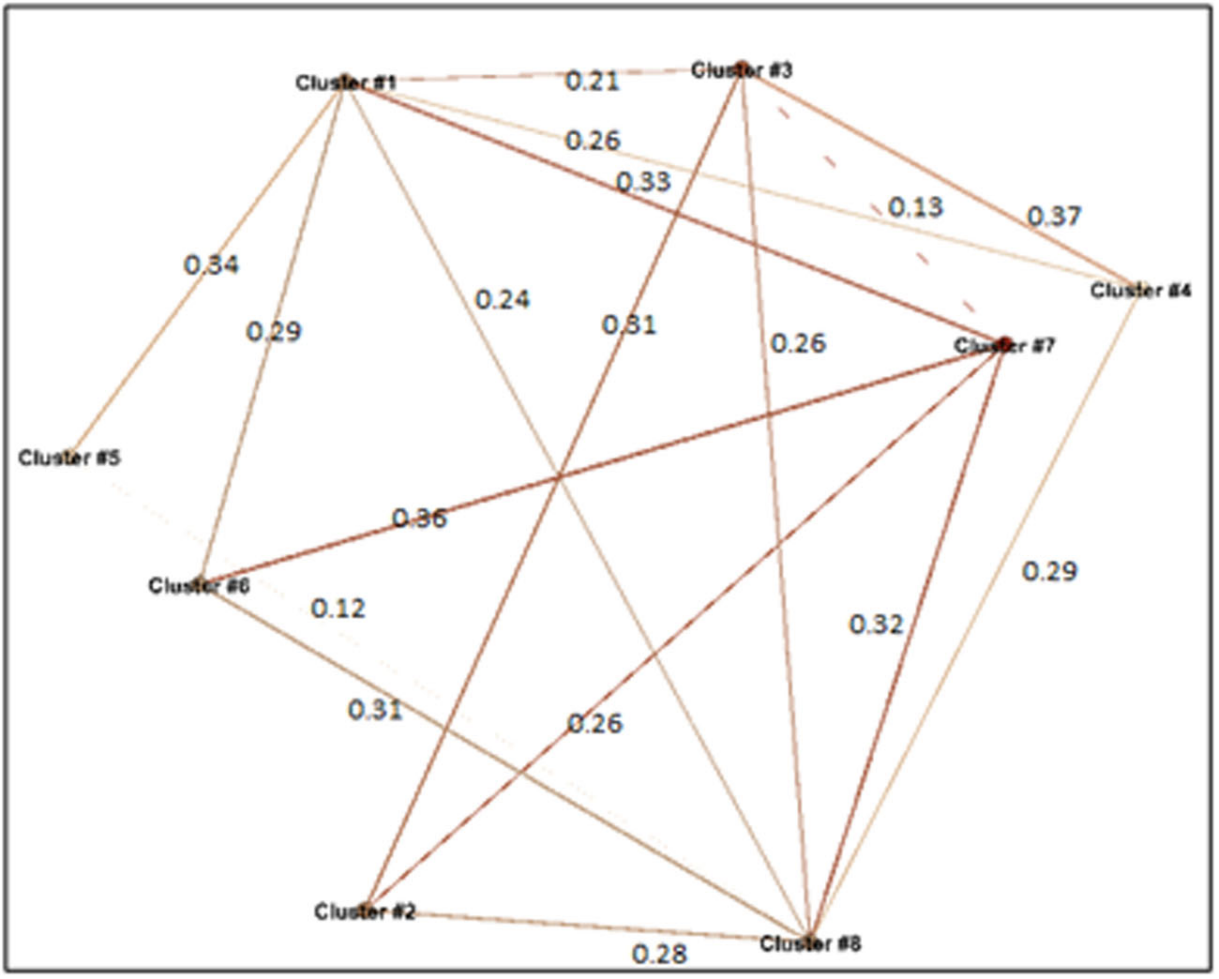
Relationship between clusters in post‐COVID‐19 studies.

At this phase, the results were classified into eight clusters (Table 4). Cluster 1 receives the highest weight (weight = 0.78), focuses primarily on the mental and psychological aspects of resilience, which have gained significant attention since the onset of COVID‐19. The results also showed that individual resilience, crisis management plans, crisis response, simulation, and models (clusters 3 and 4) have received the least weight in studies since the pandemic began. Additionally, a strong correlation was revealed between clusters 3 and 4 (w = 0.37; Figure 4).

**Table 4:** Clustering and weighting of findings from text mining after COVID‐19.

**Cluster 1, Weight: 0.78** − Baseline emotional distress − Anxiety emotional distress − Psychological resilience intervention − Mental health − Mental resilience − Moral resilience − Anxiety score − Psychological stress − Mood disorders − Moral distress − Psychological stress − Psychiatric disorders − Psychiatric medications − Insomnia − Psychological resilience intervention − Social mental resilience − Pre‐existing psychiatric	**Cluster 2, Weight: 0.46** − Emergency support − Emergency support tools − Emergency communication − Emergency management − Emergency preparedness − Emergency preparedness measures
**Cluster 3, Weight: 0.33** − Crisis management plan − Crisis management response − Crisis response model	**Cluster 4, Weight: 0.3** − Crisis simulation − Simulated crisis scenarios − Resource utilization rate
**Cluster 5, Weight: 0.53** − Health Care Workers (HCW) − Exposed HCW survivors − Unexposed HCW − Exposed HCW − HCWs' resilience − Healthcare providers' resilience	**Cluster 6, Weight: 0.42** − Resilience performance − Performance adjustment − Temporal dynamic model − Adjustment by matching − Adjustment by sustaining − Adjustment by transforming − Adjustment by extending
**Cluster 7, Weight: 0.61** − Hospital crisis committee − Hospital crisis management − Hospital disaster preparedness − Hospital disaster resilience − Hospital functionality − Hospital safety index − Hospital staff attendance − Hospital staff capability − Post‐disaster hospital resilience − Hospital system‐wide pandemic − Hospital transfusion team − Hospital‐level disaster preparedness − Hospital power system − Resilience disaster medicine	**Cluster 8, Weight: 0.35** − Disaster adaptation − Disaster management − Disaster resilience − Disaster risk reduction − Disease surveillance system

## 4 DISCUSSION

After any disaster, hospitals provide essential healthcare services for the health of population, so they must maintain effective operations in disasters.^41^ The increasing number and impact of man‐made and natural disasters have made the concept of disaster resilience increasingly crucial.^42^ Hospital resilience in disasters refers to its capability to absorb and respond to all types of disasters while sustaining essential activities, recovering to its former state, or adapting to a new situation.^43^, ^44^ Therefore, hospitals must be prepared to handle such situations.^3^ This study aimed to look at articles about hospital resilience before and after the COVID‐19 pandemic to see how this most recent worldwide disaster has changed some of the most important parts of hospital resilience. Based on the findings of the research, the discussion section could address several key aspects:

### 4.1 Evolution of hospital resilience research

Hospital resilience was the subject of fewer studies before COVID‐19 than afterward. The study's findings also showed that prior COVID‐19, articles primarily focused on organizationally tangible aspects of organizational aspect of resiliency, such as structural resilience, rather than individual and psychological resilience. In this regard, Rogers P^45^ suggested that hospital resilience can be reached using a management strategy for prevention and mitigation, planning, reaction, and recovery. Besides, Zhong S., Clark M.^28^ proposed that the strategies should be comprehensive, including structural components, nonstructural components, emergency medical functions, and disaster management mechanisms. Also, Albanese J., Birnbaum M.^46^ stated that the definitions of “safe and resilient hospitals” must address infrastructure and cross‐cutting issues of hospital disaster preparedness, such as education and training, information sharing, institutional capacity building, project implementation, networking and knowledge management, and facilitating local and regional cooperation. Lagrange Lagrange A, Simón‐Martín M.^47^ show that sustainable microgrids with energy storage enhance hospital power resilience.

The literature revealed that before COVID‐19, hospitals focused on utilities such as communication systems, sewage systems, and gas supply systems, as well as non‐structural building components like architectural elements.^7^ Additionally, preparing plans, human resources readiness, management of communication and information systems, logistics, and finance were identified as critical for improving hospital resilience.^7^, ^48^, ^49^ Most hospital resilience components before COVID‐19 were related to the mitigation and preparedness phases of the disaster risk management cycle, While improvements in hospital resilience can also be made during the response and recovery phases.

Another weakness of the studies conducted in this field before COVID‐19 was the uncertainty of resilience indicators at the individual and organizational levels. Enhancing resilience at the individual level significantly impacts improving resilience at the organizational level. As a complex healthcare provider, the hospital comprises staff with various specialties, and fostering individual resilience helps the hospital become resilient in other areas as well. Generally, the systematic review phase before COVID‐19 highlighted the predominant focus on recognizing qualities contributing to hospital resilience, emphasizing organizational, personal, and social resilience dimensions. The shift in focus from general concepts to specific aspects like mental and psychological resilience after the onset of COVID‐19 underscores the evolving nature of hospital resilience research.

### 4.2 Impact of COVID‐19 on hospital resilience dimensions

The text mining results before and after COVID‐19 began to revealed significant changes in the emphasis on different resilience clusters. The increased weight on mental and psychological aspects post‐COVID‐19 suggests a heightened awareness of the importance of addressing these factors in enhancing hospital resilience during disasters and crises. According to the results, the number of studies conducted on hospital resilience after COVID‐19 was significantly higher. In addition, the study's results after the COVID‐19 phase showed that psychological dimensions gained more weight than other clusters. Furthermore, Achour, Elhaj and Ali^50^ study on the resilience of the hospital against extreme events from the perspective of staff attendance showed that, despite the considerable amount of research on preparedness, hospitals remained vulnerable and often unable to respond effectively due to factors such as infrastructure damage and staffing shortages.

Few other studies have also noticed the importance of human resources in the resiliency of hospitals. Georgieva, Kostadinov and Semerdjieva^51^ outlined the personal and knowledge aspects of the medical specialists in the hospital as one of the dimensions of hospital resilience and also found that an inadequate understanding of the necessary adaptations to medical specialists' activities during a disaster can have a detrimental effect on a hospital's resilience. Jolgehnejad, Kahnali and Heyrani^52^ mentioned that staff, infrastructure, management, and logistics are also key components of hospital resilience. In contrast, Fallah divided the dimensions of the hospital's resilience into three categories: constructive resilience, infrastructural resilience, and administrative resilience, where individual and psychological dimensions were not considered.^53^

Naidoo^54^ also suggests that to enhance hospital resilience to future disasters, it's essential to implement multi‐pronged policies across all hospital readiness domains and at various levels. This finding underscores the importance of a comprehensive approach to bolstering hospital readiness to increase hospital resilience.

Moitinho de Almeida, Van Loenhout^54^ classified individual resilience into safety, meaningfulness, and a sense of belonging. They explained that the intricate network of anticipated and evolving adaptations and the interdependent relationship it shares with individual resilience derived hospital resilience. Also, their research argued that hospital resilience improvement plans should reflect response flexibility and concern for the well‐being of hospital staff, which is very important for acceptable disaster response and improved resilience. Furthermore, Nazari, Movahed and Soltaninejad^55^ stated that improving the continuing education program is very important to improve hospital resilience, as well as psychological support, planning, and decision‐making. Finally, improving staff awareness, education, and training about disaster‐related plans is necessary.^56^

The hospital resilience now differs from traditional disaster preparedness concepts. In disaster preparedness, hospitals plan for a relatively limited set of operational challenges, and the responses are often algorithmic. However, it is impossible for a hospital to plan a response in advance for every potential operational challenge. The factors that create resilient hospitals are not well understood. A more nuanced understanding of being a resilient hospital will provide new strategies for building resilience for future pandemics and disasters.^12^

### 4.3 Practical recommendations

Drawing from the identified themes and clusters, practical recommendations can be proposed to enhance hospital resilience in the face of future crises. These recommendations include strengthening mental health support for healthcare professionals, improving crisis management plans, and fostering a culture of teamwork and interpersonal resilience within healthcare settings.

a) Strengthening mental health support and well‐being: Given the increased focus on mental and psychological aspects of resilience post‐COVID‐19, healthcare organizations should prioritize the well‐being of their staff. Burnout, stress, and emotional strain have been prevalent among frontline workers, highlighting the urgent need to prioritize mental health support. Enhancing resilience through interventions like counseling services, peer support programs, and mental health resources can help mitigate the long‐term impact of crises on healthcare staff.
b) Improving and adapting disaster management plans: Hospitals should review and enhance their crisis management plans to better prepare for future disasters. This includes conducting regular drills and simulations to test the effectiveness of response strategies, identifying gaps in the existing plans, and updating protocols based on lessons learned from experiences and emerging research. The dynamic nature of the COVID‐19 pandemic has necessitated rapid adjustments to crisis management plans and response strategies. Lessons learned from this pandemic, such as the importance of agility, flexibility, and coordination in crisis response, can inform the development of more robust and adaptive plans to mitigate risks and ensure effective healthcare delivery during emergencies.
c) Fostering a culture of teamwork and interpersonal resilience: Building strong interpersonal relationships and teamwork among healthcare professionals is crucial for effective crisis response. Hospitals can promote collaboration through team‐building activities, cross‐training initiatives, and fostering a supportive work environment where staff feel empowered to communicate openly, share responsibilities, and work together towards common goals.
d) Investing in training and skill development: Enhancing professional skills and competencies among healthcare staff can improve their ability to adapt to challenging situations and navigate complex.
e) Sustainable resilience strategies: As healthcare systems face uncertainties and evolving threats, it is important to develop sustainable resilience strategies for future disasters. By implementing the practical recommendations derived from the study findings, hospitals can lay the foundation for a resilient healthcare system that prioritizes the mental well‐being of its workforce and ensures continuity of care under challenging conditions.

By contextualizing the study results within the current health scenario of COVID‐19 challenges, healthcare organizations can collect valuable insights on enhancing resilience, adapting to changing circumstances, and preparing for future disasters with greater confidence.

### 4.4 Implications for future research and practice

The findings underscore the need for a more comprehensive understanding of hospital resilience, especially in our disaster‐prone era. Future research could integrate insights from both pre‐and post‐pandemic studies to develop holistic resilience strategies that encompass organizational, personal, and social dimensions.

### 4.5 Limitations

The main limitations of this study included the inability to access a few full‐text articles, potentially excluding relevant information. The focus on English‐language papers during text mining may have resulted in missing insights from non‐English publications, potentially overrepresenting perspectives on hospital resilience from developed countries and neglecting issues in less developed ones. Additionally, this limitation might have reduces the geographical diversity of the included papers, thereby restricting the scope and perspectives on hospital resilience. This particularly relevant in contexts with different healthcare infrastructures and challenges, such as in less developed healthcare settings, that may still struggle with basic structural resiliency problems.

## 5 CONCLUSIONS

This research showed that after the COVID‐19, hospital resilience has attracted increased academic attention, considering the bnumber of articles published before and after this pandemic. Before COVID‐19, the focus on hospital resilience in disasters was more on reducing structural, non‐structural, and functional vulnerability. However, after the onset of COVID‐19 pandemic, greater emphasis was placed on the personal dimensions of hospital resilience particularly the mental and psychological resilience of healthcare personnel. Consequently, healthcare policymakers should prioritize these aspects of hospital resilience and develop strategies to enhance the individual and psychological resilience of hospital staff, especially medical personnel.

## AUTHOR CONTRIBUTIONS

**Golrokh Atighechian**: Conceptualization; writing—original draft; formal analysis; data curation; funding acquisition; resources. **Alireza Rahimi**: Supervision; project administration. **Mohammad Sattari**: Software; formal analysis; data curation; methodology; visualization. **Mahan Mohammadi**: Investigation; funding acquisition; writing—original draft; conceptualization; validation; writing—review and editing; supervision; data curation.

## CONFLICT OF INTEREST STATEMENT

The authors declare no conflict of interest.

## TRANSPARENCY STATEMENT

The lead author Mahan Mohammadi affirms that this manuscript is an honest, accurate, and transparent account of the study being reported; that no important aspects of the study have been omitted; and that any discrepancies from the study as planned (and, if relevant, registered) have been explained.

## Data Availability

The data that support the findings of this study are available from the corresponding author upon reasonable request.

## APPENDIX 1

Characteristics of selected papers in the systematic review phase.

**Table:**

Row	Country	Author(s)	Disaster type	Study objective	Method	Dimensions (measures) of hospital resilience	Concept of resilience
1	Australia	Zhong SH^34^	Multiple kinds of disasters, including natural disasters, manmade disasters, pandemics, and so forth	To develop a comprehensive framework of key indicators of hospital resilience.	Three‐round online modiﬁed Delphi exercise	− Hospital safety − Disaster leadership and cooperation − Disaster plans − Disaster stockpiles and − logistics management − Emergency staff − Emergency critical care − capability − Procedures − Recovery and adaptation − Mechanism	The hospital's ability to resist, absorb, and respond to the shock of disasters while maintaining its critical healthcare functions and then recover to its original state or adapt to a new one
2	Australia	Zhong SH^25^	Bioterrorism, Chemical, biological, Radiological or nuclear mass casualty event	To deﬁne hospital resilience, build a preliminary conceptual framework, and highlight possible approaches to measurement.	Literature review on Major health electronic databases	Safety and security, recovery, adaption, medical continuity and surge capacity, emergency medicine, logistic management, available resources, crisis communication, and community connectedness.	Hospital resilience is a comprehensive concept that includes structural components, nonstructural components, Emergency medical functions, and disaster management capacity
3	Malaysia	Samah AB^26^	Floods, landslides, storms and mass casualty incident	To share the RHI/BRC initiatives in investigating health infrastructure's robustness through resiliency for health infrastructure, focusing on the criteria of robustness, redundancy, and rapidity.	Hazard Mapping, Vulnerability and Capacity by the workshop participants that focus on analyzing the relationship between hazard and hospital vulnerability, capability	− Planning and Layout − Site and access − The building − Water supply system − Electrical Supply − Communication system − Gas supply system	Possible catastrophic consequences to avoid any unexpected hospital response and ad‐hoc planning
4	Iran	Motevali S.^40^	All hazard	To suggest a mixed sustainability‐resilience framework for evaluating HISsin order to enhance their performance	case study	Environmental: natural resources saving, E‐wastes generation reduction, ISO‐14040, ISO‐14001. Economic: operational cost reduction, error reduction, cycle time reduction development, Maintenance software cost reduction, data integration. Social: Trust, health and well‐being, education and training, satisfaction, sense of belonging, job security, job opportunity	Resilience as a new concept can be used for better responding to random accidents in diﬀerent industries in the face of disruptions, organizations can come back to the normal state by incorporating resilience strategies
5	Malaysia	Samsuddi N.^27^	Flood, computer system failure, power outages	To investigate the hospitals' preparedness attributes and resilience indicators	Cross‐sectional	Ability to adapt and recover in a timely manner. Ability to identify health facilities or health system problems. Ability to mobilize resources. Ability to meet healthcare priorities and achieve essential functions. Ability to reduce the probability of building failures. Ability to reduce the probability of failures in redundant systems	Resilience (i.e., robustness, redundancy, resourcefulness, and rapidity)
6	China	Zhong SH^28^	Infectious disease, mass casualty incidents, career poising and food poisingbioterrorism, and nuclear terrorism	To explore the resilience status among tertiary hospitals in Shandong Province, China	Cross‐sectional: Analysis of existing literature and through a three‐round modified Delphi. The questionnaire was extracted	(1) Hospital demographic data, (2) Hospital internal safety.(3) Emergency leadership and cooperation, (4) Disaster plan, (5) Disaster stockpile and logistics management, (6) Emergency staff, (7) Emergency critical care capability, (8) Training and drills, and (9) Disaster recovery mechanism	Including prevention, preparedness, responsiveness, and recovery and adaptation phases). It also seeks to achieve the goal of improving hospital predisaster strength (robustness) and promoting rapidity of response and recovery
7	Malaysia	Takim R.^35^	Flood fire ceiling collapses unhygienic hospital conditions stolen hospital equipment technical glitch	To evaluate the content validity of the disaster resilience hospital assessment instrument	Two document analyzes (seven instruments) and CVR & CVI questionnaires (six experts)	Structural: Presence of ramps built with fire resistive. Nonstructural: Condition and safety of telecommunication system, medical gas system, fuel storage, fire suppression system, Supplies in the laboratory, pharmacy, and general stores. Functional: Condition of hospital access routes Reasonably free from environmental hazards condition of internal emergency access routes and proper zoning of the service area	A disaster‐resilient hospital can resist, absorb, and respond to the shock of disasters while still retaining its essential functionality
8	The USA	Bruneau M.^36^	Earthquakes	To provide a framework for future research to determine the resiliency of different units of analysis and systems	A series of analytical steps	Technical: The ability of physical systems. Organizational: The capacity of organizations that manage critical facilities and have the responsibility for carrying out critical disaster‐related functions. Social: To lessen the extent of suffering caused by earthquakes in communities and governmental jurisdictions. Economic: The capacity to reduce both direct and indirect economic losses	The ability to recover quickly from illness, change, or misfortune
9	Singapore	Chan A. O. M.^38^	Work‐related traumatic incidents and crises	To examine exposure to work‐related crises and the resiliency of healthcare workers in public hospitals in Singapore	Using a pilot 5‐point Likert questionnaire	Resiliency can be acquired through mental health training, improving the individual's self‐confidence and self‐efficacy	The ability to cope with situations not within the individual's control, the ability to adapt to change, and the ability to bounce back
10	China	Zhong SH^28^	All disasters	− To develop a preliminary evaluation framework − To extract key component − To examine the construct validity and internal consistency − To establish scoring models to measure the level of hospital disaster resilience	A scoping review and Modified‐Delphi consultation.	Eight key domains were identified: hospital safety, command, communication and cooperation system, disaster plan, resource stockpile, staff capability, disaster training and drills, emergency services and surge capability, and recovery and adaptation	the ability of hospitals to resist, absorb, and respond to the shock of disasters while maintaining and surging essential health services
11	Iran	Azadian S.^37^	All crisis	To evaluate validity and reliability of the questionnaire to assess crisis management	Descriptive	Awareness, flexibility, acceptance of fallibility, learning culture, transparency, and management commitment	The ability of a system to reduce the chances of a shock, to absorb such a shock if it occurs, and to recover quickly after a shock
12	Iran	Khanmohammadi S.^29^	Earthquake	To promote the post‐earthquake recovery process of a hospitals	In‐depth interviews and government reports, internal publications, and scholarly articles.	Endogenous variables: patients in backlog, functional capacity, patients in hospital, medicine inventory, …Exogenous variables: earthquake intensity, resources to allocate, patient arrival rate, technical systems and medical equipment. Excluded variables: transportation serviceability, condition of supporting lifelines, performance of other hospitals in the region	Hospitals (i.e., their ability to resist, absorb, and respond to the shock of an earthquake while providing the required essential health services
13	Lebanon, Liberia, and Indonesia	Kruk M. E.^30^	Population influx in Leba nonsevere infectious disease outbreak in Liberia anticipated disaster shocks in Indonesia	Assessment of countries' progress in building resilience and indicate areas for action	Three case studies	Aware: Knowing risks. Diverse: Effectively respond to a range of health needs. Self‐regulating: Isolate threat and maintain core functions. Integrated: Coordinate with nonhealth actors (education, transport, police, media, private enterprise), Engage citizens and communities to build trust. Adaptive: Shift resources to meet the needs	The capacity of health actors, institutions, and populations to prepare for and effectively respond to crises maintain core functions when a crisis hits
14	The USA	Mallak L. A.^39^	Natural disasters	Nursing staff in emergency situations	Conﬁrmatory factor analysis	Goal‐directed solution seeking, avoidance, critical understanding, role dependence, source reliance, and resource access	Resilience is the ability of an individual or organization to rapidly design and implement positive adaptive behaviors matched to the Immediate situation while enduring minimal stress
15	Canada	Khan Y.^31^	Noveland re‐emerging infectious diseases wildfires	To describe the essential elements of a resilient public health system and how the elements interact as a complex adaptive system	A qualitative design employing the Structured Interview Matrix facilitation technique in six focus groups	Governance and leadership planning process collaborative networks community engagement risk analysis surveillance and monitoring practice and experience resources workforce capacity communication learning and evaluation	Adaptability during emergency response, and in preparing for future events
16	The USA	Cimellaro G.P.^32^	Earthquake	To identify trend leads toward the deﬁnition of complex recovery models that are able to describe the process over time and the spatial deﬁnition of recovery (e.g. meta‐models for the case of health care facilities)	Case study	Intensity measures: Ground motion hazard estimation, laboratory experiments, observations on past earthquakes, performance requirements. Response parameters: (interstory drift, absolute acceleration, and …): damage state estimation, response estimation. Performance measures (with associated damage limits): fragility analysis, performance limit state estimation. Recovery estimation, loss estimation	The ability of a system to reduce the chances of a shock, absorb such a shock if it occurs and recover quickly after a shock
17	The USA	Somers S.^33^	Complex situations	To challenge this orthodox view and suggests a new paradigm, one that focuses on creating organizational structures and processes that build organizational resilience potential	Case study	Level of perceived risk managerial information seeking organizational structure continuity planning participation in community planning department accreditation	For example, resilience has been seen as the ability of the organization to simply ‘bounce back' from a ‘distinctive, discontinuous event that creates vulnerability and requires an unusual response
